# Molecular landscape of methicillin-resistant *Staphylococcus aureus* strains in clinical infections from hospitals in Lagos, Nigeria

**DOI:** 10.1093/jacamr/dlaf161

**Published:** 2025-09-19

**Authors:** Adesola Olalekan, Sébastien Boutin, Christopher Mark Watson, Luiza Galarion, Solayide Adesida, Bamidele Iwalokun, Olayiwola Popoola, Seraphine Esemu, Richard Adegbola, Dennis Nurjadi

**Affiliations:** Department of Medical Laboratory Science, College of Medicine, University of Lagos, Lagos, Nigeria; Institute of Medical Microbiology, University of Lübeck and University Hospital Schleswig-Holstein Campus Lübeck, Ratzeburger Allee 160, Lübeck 23562, Germany; German Centre for Infection Research (DZIF), Partner Site Hamburg-Lübeck-Borstel-Riems, Lübeck, Germany; Airway Research Centre North (ARCN), German Centre for Lung Research (DZL), Lübeck, Germany; Leeds Institute of Medical Research, University of Leeds, St. James’s University Hospital, Leeds, UK; North East and Yorkshire Genomic Laboratory Hub, Central Lab, St. James’s University Hospital, Leeds, UK; Faculty of Biological Sciences, School of Molecular and Cellular Biology, University of Leeds, Leeds, UK; Department of Microbiology, Faculty of Science, University of Lagos, Akoka, Lagos, Nigeria; Molecular Biology & Biotechnology Department, Nigerian Institute of Medical Research, Lagos, Nigeria; Department of Medical Laboratory Science, College of Medicine, University of Lagos, Lagos, Nigeria; Department of Microbiology and Parasitology, Faculty of Science, University of Buea, Buea, Cameroon; Molecular Biology & Biotechnology Department, Nigerian Institute of Medical Research, Lagos, Nigeria; Institute of Medical Microbiology, University of Lübeck and University Hospital Schleswig-Holstein Campus Lübeck, Ratzeburger Allee 160, Lübeck 23562, Germany; German Centre for Infection Research (DZIF), Partner Site Hamburg-Lübeck-Borstel-Riems, Lübeck, Germany

## Abstract

**Background and objectives:**

Multidrug-resistant *Staphylococcus aureus* accounts for a significant proportion of antimicrobial resistance (AMR)-associated infections worldwide. This study investigated the molecular profile of MRSA in Nigeria, providing valuable genomic data to fill existing knowledge gaps and highlighting its importance in the context of the global AMR crisis.

**Methods:**

A total of 107 isolates were obtained from patient samples, including wound swabs/pus (65 isolates, 60.7%), blood cultures (16 isolates, 15%), urine/urinary catheter (8 isolates, 7.5%) and other sources. Species identification was performed using MALDI-TOF, and antimicrobial susceptibility testing was performed using the VITEK^®^2 system. Genomic DNA was extracted and subjected to whole-genome sequencing using short-read Illumina technology. In addition, a subset of isolates underwent long-read sequencing using Oxford Nanopore technology.

**Results:**

Among the 107 isolates, 63 (59%) were identified as MRSA, with 58 (92%) carrying the *mecA* gene. The MRSA isolates exhibited high resistance to non-β-lactam antibiotics, particularly trimethoprim/sulfamethoxazole (95.3%), erythromycin (76.6%), gentamicin (71.4%) and quinolones (69.8%). The most prevalent MRSA belonged to the Bengal Bay clone [t657/ST772/Staphylococcal Cassette Chromosome *mec* (SCC*mec*) V(5C2)/Panton-Valentine leukocidin (PVL) + MRSA], followed by t4690/ST152/SCC*mec* Vc(5C2&5)/PVL + MRSA and ST8 (t008, *n* = 1; t064, *n* = 4)/SCC*mec* Vc(5C2&5). Phylogenetic analysis suggests both community-associated transmission and possible importation of strains.

**Conclusions:**

This study highlights the significant burden of MRSA in Nigeria, with the high-risk Bengal Bay MRSA clone as the most common strain. The widespread resistance to non-β-lactam antibiotics underscores the urgent need for enhanced surveillance, infection control and antibiotic stewardship to mitigate its spread.

## Introduction

Methicillin-resistant *Staphylococcus. aureus* (MRSA) represents a critical global health threat, recognized as the leading cause of community-associated and healthcare-acquired infections across both low- and middle-income countries (LMICs) and high-income countries.^1^ MRSA is classified by the World Health Organization (WHO) as one of the high-priority pathogens due to its capacity to cause persistent and severe infections, such as soft tissue infections,^2,3^ often resulting in high mortality and morbidity. According to a systematic review, multidrug-resistant (MDR) *S. aureus* accounts for approximately 50% of the global burden of infections attributable to antimicrobial resistance (AMR),^4^ highlighting the significance of this pathogen in the global AMR crisis. Effective surveillance is essential for tracking the diversity of *S. aureus* clones and monitoring the emergence and spread of high-risk MRSA clones. Such efforts often involve advanced molecular methods, including *spa*-typing, multi-locus sequence typing (MLST), and Staphylococcal Cassette Chromosome *mec* (SCC*mec*) typing.^5^ However, high-resolution molecular epidemiological data remain sparse across many African countries, including Nigeria, where MRSA prevalence rates and clonal composition exhibit substantial variability.^6,7^ The heterogeneity and dynamic evolution of the MRSA and other bacterial populations in Africa, partly driven by travel and migration, further complicate the implementation of effective intervention.^7,8^

Despite these challenges, Nigeria lacks detailed data on the molecular epidemiology and resistance mechanisms of *S. aureus*. Current studies^9–11^ rarely employ WGS, a critical tool for elucidating genetic diversity, resistance pathways and transmission dynamics. Notably, MRSA clones such as CC80-MRSA-IV, ST772-MRSA-V (‘Bengal Bay Clone’) and ST8-MRSA-IVa (‘USA300 clone’), which are globally recognized high-risk clones,^12–14^ have been reported in other regions but remain underexplored in the Nigerian context. This gap hinders the development of evidence-based infection control measures and antimicrobial stewardship programmes. In addition, non-β-lactam antibiotics, such as trimethoprim/sulfamethoxazole, are commonly used to treat *S. aureus* infections.^15^ Further, this trimethoprim/sulfamethoxazole is considered one of the most important drugs in treating or preventing the onset of HIV-related infections.^16^ However, resistance to trimethoprim/sulfamethoxazole is more frequently reported in Africa than in developed countries.^17^ Therefore, it is crucial to investigate the specific mechanisms underlying resistance to trimethoprim/sulfamethoxazole.

Lagos, a cosmopolitan hub and economic centre with strong ties to Asia (particularly India and China) and Europe, offers a unique setting for examining the genetic and phenotypic diversity of MRSA. Our study aims to fill in existing knowledge gaps, providing valuable insights into MRSA characteristics in Nigeria and supporting broader efforts to combat AMR across Africa.

## Materials and methods

### Ethical considerations

We obtained ethical approval for this study from the Health Research Ethics Committee of the College of Medicine, University of Lagos (CMUL/HREC/05/17/136).

### Study sites and isolates collection

Between February and August 2017, a total of 107 clinical isolates of *S. aureus* were collected from the Microbiology Laboratories of three major teaching and specialized hospitals: National Orthopaedic Hospital (NOHL), Igbobi (Hospital A); Lagos State University Hospital (LASUTH), Ikeja (Hospital B); and Lagos University Hospital (LUTH) (Hospital C) (Figure S1, available as Supplementary data at JAC-AMR Online). All hospitals are located in the urban city of Lagos State, Nigeria. The isolates were recovered from outpatients or patients upon admission to the hospital and sample types: wound swab/pus [*n* *=* 65 (60.7%)], blood culture [*n* *=* 16 (15%)], urine/urinary catheter [*n* *=* 8 (7.5%)] and high vaginal swab/endocervical swab [*n* *=* 4 (3.7%)]. Key information about the isolates (including source of sample and sex of the patients) was recorded (Table S1). A convenience sampling technique was used, and only the available *S. aureus* isolates from the laboratories were included, which may not accurately represent all *S. aureus* isolates obtained during the study period. The isolates included in the final analysis were non-repetitive, i.e. only one isolate per patient was included.

### Species identification and antibiotic susceptibility testing

The identification of *S. aureus* was performed using MALDI-TOF/MS on a Microflex LT instrument (Bruker Daltonik GmbH, Bremen, Germany). Antibiotic susceptibility testing (AST) was conducted using a VITEK^®^2 automated system version 08.01 to determine the antibiotic susceptibility profiles and MICs, interpreted according to the EUCAST clinical breakpoints version 9.0 (January 2019). S*. aureus* ATCC^®^25923 was used for quality control.

### WGS and sequence analysis

All 107 *S. aureus* isolates in this study were subjected to WGS. Genomic DNA extraction was carried out according to the protocol available at https://microbesng.com and as previously described.^18^ Briefly, library preparation was performed using the Nextera XT Library Prep Kit (Illumina, San Diego, USA), following the manufacturer’s guidelines, with modification: the input DNA was doubled, and the PCR elongation time was extended to 45s. DNA quantification and library preparation were automated using a Hamilton Microlab STAR liquid handling system (Hamilton Bonaduz AG, Switzerland). Pooled libraries were quantified using the Kapa Biosystems Library Quantification Kit for Illumina. The libraries were then subjected to sequencing on the Illumina platform (HiSeq/NovaSeq) using a 250 bp paired-end protocol.

Additionally, genomic DNA for Oxford Nanopore Technology (ONT) was extracted utilizing the Sigma-Aldrich Bacterial Genomic DNA Kit and purified through elution using the Monarch PCR and DNA clean-up kits. Long-read libraries were prepared with the ONT Rapid Sequencing Kit SQK-RAD004 (ONT, UK), starting with 400 ng of genomic DNA. Barcoded samples were pooled into a single sequencing library and loaded onto a FLO-MIN106 (R.9.4.1) flow cell in a MinION device (ONT, UK). Raw reads from Nanopore sequencing were curated using filtlong (https://github.com/rrwick/Filtlong, v0.2.1 with the parameters –min-length 2500, –keep percent 95). The curated reads were used to generate hybrid assembly with Unicycler (v0.5.0). Illumina reads were re-mapped to the hybrid draft genome using bwa (v0.7.17-r1188) to polish the genome using Polypolish (v0.5.0). Assembly quality was assessed using QUAST (v5·0·2). Species identification and contamination screening were conducted using Mash (sub-command screen), comparing each draft genome to a reference database composed of a representative genome of each species present in the NCBI Microbial Genomes resource (https://www.ncbi.nlm.nih.gov/genome/microbes/). Further genotyping, including spa typing, mec cassette typing and agr group assignment, was conducted using Bactopia^19^ (v2.2.0, sub-programmes; spatyper v0.3.3 and agrVATE v1.0.2).

To identify antimicrobial resistance genes (ARGs), virulence factors and plasmid replicon types, the complete draft genomes were processed through available databases using Abricate (v 1.0.1 https://github.com/tseemann/abricate) (parameters: -minid 90 -mincov 80), for identification of AMR (NCBI, CARD, ARG-ANNOT, ResFinder, MEGARES databases), virulence factors (VFDB) and plasmid type (PlasmidFinder database) to determine the Inc type of the plasmid.^20–26^ The core genome was evaluated using Roary (v 3.13.0) for genes present in more than 99% of the population as core genome.^27^ The core genome was then processed through Gubbins (v 3.2.1) to correct for recombination events.^28^ In the end, 62 607 polymorphic sites were used to construct the phylogenetic tree with Raxml (v 8.2.12).^29^

To compare our ST772 isolates to publicly available *S. aureus* ST772 genomes from human hosts, we used the data available on the NCBI microbial genomes resources and selected all the isolates from human hosts, with a geographical location available in the metadata and belonging to the ST772. We obtained 375 public genomes and compared them to our isolate usage average nucleotide identity (ANI) with the software aniclustermap (https://github.com/moshi4/ANIclustermap). The newick format dendrogram from the software was used to display the genomic identity.

### Data availability

The raw sequence data generated in this study are available on the NCBI database with accession number PRJNA1235457.

### Statistical analysis

The distribution of categorical data was analysed using Fisher’s exact test, with a *P* value of <0.05 considered statistically significant. All descriptive statistics were performed using STATA, version 18 (StataCorp LLC).

## Results

A total of 107 isolates of *S. aureus* were collected for this study from three specialist and teaching hospitals: Hospital A (*n* = 46, 43%), Hospital B (*n* = 22, 20.6%) and Hospital C (*n* = 39, 36.4%). The sampling sites are illustrated in Figure S1.

### Molecular characteristics and antibiotic resistance patterns of MRSA and MSSA across study sites

Based on the phenotypic AST results, 63 (59%) isolates were identified as MRSA and 44 (41%) as methicillin-susceptible *Staphylococcus aureus* (MSSA). The proportion of MRSA at Hospital A was the highest, with 71.7% (33/46), compared with Hospital B (31.8%; 7/22) and Hospital C (59%/23/39). Most MRSA isolates also exhibited a high prevalence of resistance towards non-beta-lactam antibiotics with trimethoprim/sulfamethoxazole being the highest at 95.3% (60/63), followed by erythromycin with 76.6% (47/63), gentamicin with 71.4% (45/63) and quinolones with 69.8% (44/63) (Table S2). For MSSA, resistance to non-beta-lactam antibiotics is highest for trimethoprim/sulfamethoxazole with 52.3% (23/44) and tetracycline with 45.5% (20/44). Using the definition of phenotypic resistance to three or more independent classes of antibiotics as MDR, 93.7% (59/63) of the MRSA isolates collected in our study were classified as MDR, which was significantly higher than the 29.6% (15/44) of MSSA isolates classified as MDR (*P* < 0.001). A comparison of selected ARGs, antibiotic susceptibility and the presence of virulence factors is summarized in Table 1.

**Table 1.** Comparison between molecular and phenotypic characteristics of MRSA and MSSA isolates

	MRSA (*n* = 63)		MSSA (*n* = 44)		Total (*n* = 107)		*P* value^a^
	Number	%	Number	%	Number	%	
Antibiotic resistance genes							
*mecA*^b^	58	92.06	0	0	58	54.21	<0.001
*blaZ*	63	100	37	84.09	100	93.46	0.001
*msr(A)-*like	43	68.25	0	0	43	40.19	<0.001
*mph*(C)	39	61.9	0	0	39	36.45	<0.001
*erm*(B)	0	0	2	4.54	2	1.87	0.2
*erm(*C)	2	3.17	2	4.54	4	3.74	1
*aac*(6′)-*aph*(2″)	45	71.43	9	20.45	54	50.47	<0.001
*ant(*6)-Ia	42	66.67	10	22.73	52	48.6	<0.001
*aph*(3′)-III	42	66.67	11	25	53	49.53	<0.001
*Str*	4	6.35	0	0	4	3.74	0.1
*aadD*-like	3	4.76	2	4.54	5	4.67	1
*dfrG*	60	95.24	35	79.54	95	88.79	0.03
*tet*(M)	0	0	2	4.54	2	1.87	0.2
*tet*(K)	18	28.57	15	34.09	33	30.84	0.7
*tet*(L)	0	0	4	9.09	4	3.74	0.03
*cat*(pC221)	4	6.35	3	6.82	7	6.54	1
*lnu*(A)-like	1	1.59	1	2.27	2	1.87	1
Antibiotic susceptibility							
Benzyl penicillin	63	100	38	86.36	101	94.39	0.004
Oxacillin	61	96.83	0	0	61	57.01	<0.001
Inducible clindamycin resistant	2	3.17	2	4.54	4	3.74	1
Clindamycin	7	11.11	4	9.09	11	10.28	1
Erythromycin	47	74.6	4	9.09	51	47.66	<0.001
Gentamicin	45	71.43	9	20.45	54	50.47	<0.001
Levofloxacin	44	69.84	7	15.91	51	47.66	<0.001
Tetracycline	24	38.1	23	52.27	47	43.93	0.2
Trimethoprim/sulfamethoxazole	60	95.24	20	45.45	80	74.77	<0.001
Rifampicin	4	6.35	0	0	4	3.74	0.1
Fosfomycin	6	9.52	0	0	6	5.61	0.04
Fusidic acid	3	4.76	0	0	3	2.8	0.3
Daptomycin	2	3.17	0	0	2	1.87	0.5
Multi-locus sequence type							
ST772	36	57.14	0	0	36	33.64	<0.001
ST152^c^	7	11.11	8	18.18	15	14.02	0.4
ST508	1	1.59	6	13.64	7	6.54	0.02
ST8	5	7.94	2	4.55	7	6.54	0.7
ST30	0	0	7	15.91	7	6.54	0.001
ST121	1	1.59	5	11.36	6	5.61	0.08
ST789	3	4.76	3	6.82	6	5.61	0.7
ST1	2	3.17	3	6.82	5	4.67	0.4
ST15	1	1.59	4	9.09	5	4.67	0.2
ST5	2	3.17	2	4.55	4	3.74	1
ST7669	3	4.76	0	0	3	2.8	0.3
ST88	2	3.17	0	0	2	1.87	0.5
ST94	0	0	2	4.55	2	1.87	0.2
ST2434	0	0	1	2.27	1	0.93	0.4
ST669	0	0	1	2.27	1	0.93	0.4
Virulence factors							
PVL (*lukF*/*lukS*)	50	79	25	57	75	70	0.01
TSST-1	1	2	1	2	2	1.8	0.8

PVL, Panton-Valentine leukocidin; ST, sequence type; TSST, toxic shock toxin; MRSA, methicillin-resistant *S. aureus*; MSSA, methicillin-susceptible *S. aureus*.

^a^ *P* values were calculated using the Fisher’s exact test.

^b^ *n* = 5 MRSA did not harbour a *mec*-gene.

^c^Including ST152-1LV (*n* = 3) and ST152 (*n* = 5).

### Molecular characteristics of MRSA isolates (SCC*mec*, MLST typing and virulence markers)

Of the 63 MRSA isolates in our study, 58 (92%) harboured the *mecA* gene. Only 56 carried typable SCC*mec*. The most common types were SCC*mec* V (5C2), 37 (66%); Vc (5C2&5), 14 (25%); V(5C2&5), 3 (5.4%); and IVa (2B), 2 (3.6%). Thirty-six (57%) belong to ST772 (including a single allele variant of ST772-1LV) with the *spa*-type t657. Thirty-four (94%) of the ST772 isolates were PVL positive and harbour the SCC*mec* type V(5C2), consistent with the ‘Bengal Bay clone’ (t657/ST772/SCC*mec* V(5C2)/PVL + MRSA).

These ST772 MRSA were mainly isolated from wound swabs (23/36) and (7/36) blood cultures. In two study centres, Hospital C and Hospital A, ST772 was the predominant MRSA clone isolate with 38% (15/39) and 41% (19/46), respectively. At the study site Hospital C, only 9% (2/22) of the MRSA detected belong to ST772. The second and third most prevalent MRSA clones were t4690/ST152/SCC*mec* Vc(5C2&5)/PVL + MRSA and ST8 (comprising one t008 and four t064 isolates), respectively.

Compared with non-ST772 MRSA, isolates belonging to ST772 harboured more ARGs with a median of 26 ARGs (range 26–28) compared with 19 (range 12–26) ARGs in non-ST772 MRSA.

Regarding virulence markers, 70% (75/107) of *S. aureus* isolates harboured *lukS* and *lukF*, which encode Panton-Valentine leukocidin (PVL). A significantly higher proportion of MRSA were PVL positive (79%, 50/63) compared with MSSA (57%, 25/44), with a *P* value of 0.01 (Fisher’s exact test). The toxic shock toxin gene, *tsst-1*, was detected in one MSSA isolate from blood and one MRSA from wound sample.

The PVL-positive *S. aureus* isolates belong to ST772, ST152, ST30, ST121, ST1, ST15, ST7669 and ST88 in decreasing order of prevalence. The PVL-negative isolates belong to ST508, ST8, ST789, ST5, ST15, ST772, ST94, ST2434 and ST669, also in decreasing order. Three isolates in this study were newly assigned to the MLST sequence type ST7669.

### Potential MRSA transmission clusters

Within ST772, we identified six SNP clusters (median SNP distance ≤ 3), which may indicate transmission events. The threshold of three SNPs was decided based on the overall SNP distribution within the ST772, which shows that the next lowest SNPs distance after this threshold is 19.

**Figure 1.**
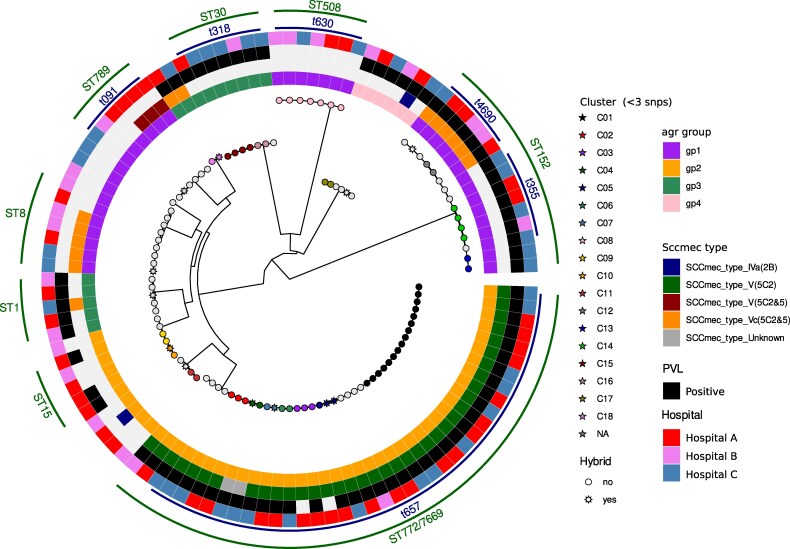
Phylogenetic tree of MRSA isolates from Nigeria. Whole-genome sequencing suggested the predominance of ST772 ‘Bengal Bay’ MRSA isolated from three different sampling sites in Nigeria. The most outer lines indicate the MLST designation, followed by *spa*-types. PVL, Panton-Valentine leukocidin; ST, sequence type (see Table S4).

The largest SNP cluster of ST772 MRSA consists of 16 isolates, collected from Hospital A and Hospital C. For ST152, we identified three potential transmission clusters. The largest cluster of potential MRSA transmissions involved two isolates of t4690/ST152/SCC*mec* Vc(5C2&5) PVL + MRSA from the study centre Hospital B. The remaining two ST152 clusters were MSSA clusters, one (Cluster C14) involved four isolates, originating from all three study sites and the other cluster (Cluster C13) (Figure 1 and Table S4).

### Comparison of Nigerian ST772 MRSA with globally circulating strains

**Figure 2.**
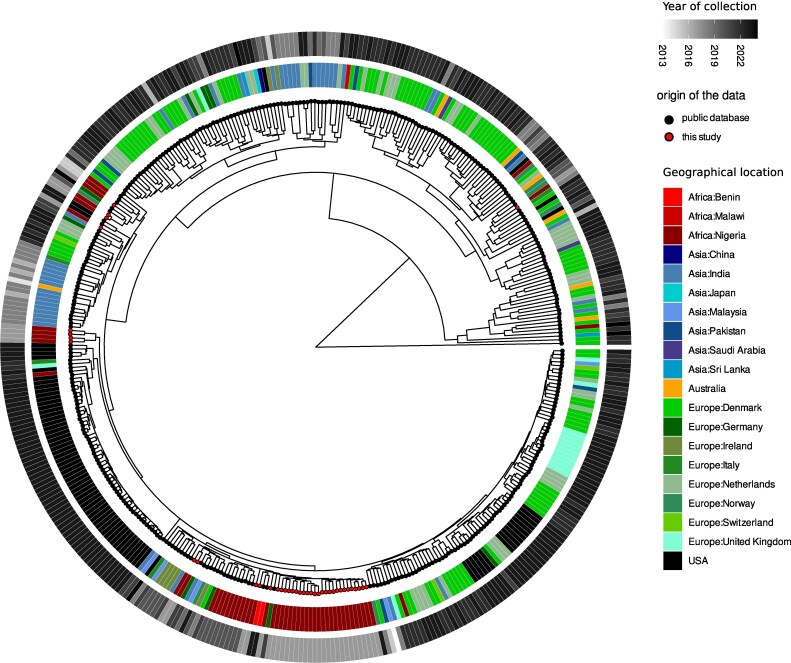
Comparative genomics of ST772 MRSA in the study to publicly available sequences. All genomes from ST772 from RefSeq were downloaded, and only the ones with complete metadata regarding host source as human and geographical location were kept. Genomic comparison was done using ANI. It appears that there is an ‘African clade’ of ST772, which is quite prominent, comprising most of the strains from this study and also from other studies, suggesting a high local prevalence or even a community reservoir in Nigeria (see Table S3).

To compare the ST772 strains collected in this study with other ST772 strains from different countries and regions, we aligned our draft genomes with 388 publicly available genomes from NCBI. Most of the published ST772 MRSA genome sequences are from human clinical isolates, with only a few from other sectors: one from animal, five from environmental samples and six from food sources (Figure 2).

Based on phylogenetic analysis using ANI, we observed that the majority of the isolates in our study clustered with other ST772 MRSA genomes from Benin and another study from Nigeria. This suggests that the African ST772 isolates may belong to the same clade. In addition, several isolates from our study clustered in clades dominated by European and Asian ST772 strains. ANI comparison table is provided in Table S3.

### Determinants of trimethoprim/sulfamethoxazole resistance

All 93 trimethoprim/sulfamethoxazole-resistant *S. aureus* isolates carried at least one trimethoprim resistance determinant. Among these, 87 isolates (93%) carried only the *dfrG* gene, while six isolates (6.5%) carried both *dfrG* and *dfrA* (also known as *dfrS1*). Interestingly, three isolates harbouring both *dfrG* and *dfrA* did not exhibit phenotypic resistance to trimethoprim/sulfamethoxazole. Next, we aimed to identify the genetic environment surrounding the *dfrG* gene, given its frequent location on transposable elements.^30^

**Figure 3.**
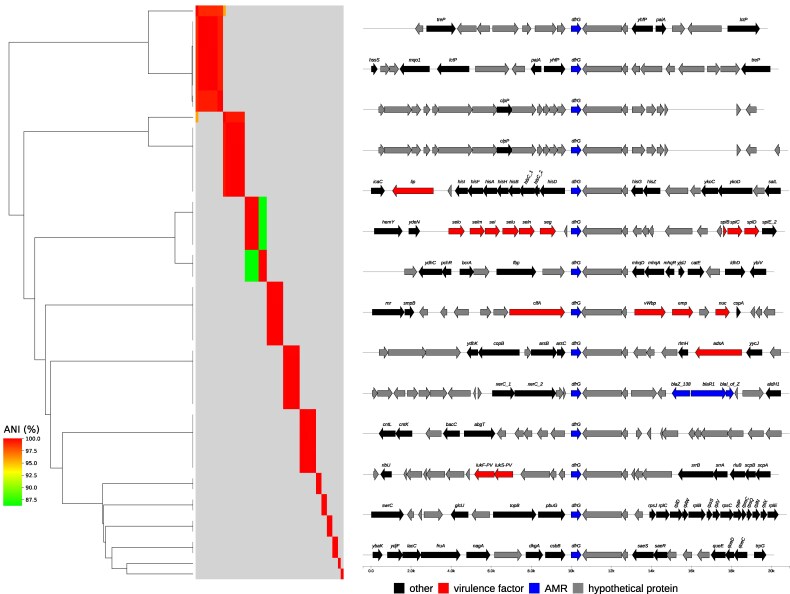
Genomic environment of the trimethoprim resistance determinant *dfrG*. Phylogeny depicting variants of *dfrG*, calculated by the ANI between the genomic context (surrounding 10 kb) of *dfrG*.

This analysis revealed 14 distinct localizations of *dfrG* among our study isolates (Figure 3). Notably, in two variants, the *dfrG* gene is situated near virulence gene clusters associated with host defences, such as the exotoxin gene cluster comprising *selo*, *selm*, *sei*, *selu*, *seln* and *seg*, and another cluster that includes clumping factor A (*clfA*), von Willebrand factor-binding protein (vWbp), extracellular matrix protein (emp) and *nuc* genes. Regarding sulfamethoxazole resistance determinants, we identified only the F_17_L and E_208_K mutations, known to be primary and secondary mutations, respectively, conferring sulphonamide resistance in *S. aureus*. Overall, seven strains carried both the primary mutation F_17_L and the secondary mutation E_208_K, all belong to ST8. Of these, five strains were MRSA with SCC*mec* Vc(5C2&5) and two were MSSA. The MIC of trimethoprim/sulfamethoxazole for all the seven isolates is ≥320, while only two isolates carried the E_208_K mutation alone; they belong to ST88/MRSA/Vc(5C2&5) and both also had a trimethoprim/sulfamethoxazole MIC of ≥320 (Figure S2).

## Discussion

The molecular epidemiological analysis identified the ST772 Bengal Bay clone as the predominant MRSA lineage in our study population. The predominance of the ST772 clone in this setting was unexpected, as it had not been previously considered a major clone circulating in Sub-Saharan Africa. This clone, characterized by the *spa*-type t657, SCC*mec* type V(5C2) and PVL positivity, is a globally recognized high-risk clone originating in South Asia.^14^ To the best of our knowledge, ST772 MRSA was rarely detected in Nigeria and was not considered one of the major circulating clones in Africa prior to 2017.^31^ However, in line with our findings, the study performed by Ogundipe *et al*.^32^ also found a substantial proportion of the MRSA found belonging to ST772 (30%, 9/30) in individuals frequenting live bird markets in Nigeria.

The abundance of ST772 in two independent studies raises important questions regarding its origin and transmission pathways within Nigeria. The emergence of the ST772 clone in Lagos may have been influenced by immigration and its potential introduction from Asia. Lagos State, as Nigeria’s economic hub, attracts a diverse population and serves as a central point for international business activity.^33^ The city’s substantial expatriate community, including a significant representation from India, where the ST772 clone is highly prevalent,^14^ may have contributed to its introduction and establishment. Genomic comparisons between ST772 MRSA isolates from this study and genomes from public repositories revealed that some ST772 MRSA isolates clustered with genomes from various countries and regions worldwide, reinforcing the hypothesis that international travel and global mobility contribute to the dissemination of this high-risk clone.^34,35^ At the same time, the clustering of certain isolates within an African clade suggests the existence of a persistent regional reservoir, further complicating the elucidation of this strain’s transmission routes. Strains clustering with those from Asia or Europe may indicate importation events from these regions. Conversely, strains clustering in the African clade may reflect a sustained reservoir within the community.

Our findings highlight the concerning levels of antimicrobial co-resistance among MRSA isolates in Nigeria. More than 93% of MRSA isolates met the criteria for multidrug resistance, a rate more than 3-fold higher than the 29.6% observed among MSSA isolates. Resistance to trimethoprim/sulfamethoxazole was most common (95.3%), followed by erythromycin (76.6%), gentamicin (71.4%) and quinolones (69.8%). These high resistance rates not only limit therapeutic options but also underscore the urgency of implementing robust antimicrobial stewardship and infection prevention strategies to curb further dissemination.

High resistance rates to trimethoprim/sulfamethoxazole have been reported repeatedly.^30,31,36,37^ These alarming resistance levels may be attributed to the widespread use of this agent in the treatment of respiratory infections and malaria, as well as the substandard quality of circulating drugs in the region.^38^ These findings underscore the urgent need for strengthened antibiotic stewardship and measures to address the circulation of substandard pharmaceuticals to combat the growing threat of AMR in Nigeria.

Both trimethoprim and sulfamethoxazole inhibit folate biosynthesis at two key steps in the biosynthetic pathway. Resistance to trimethoprim in *S. aureus* is often mediated by the production of extrachromosomal dihydrofolate reductase (DHFR) encoded by mobile *dfr* genes.^39^ Resistance to sulfamethoxazole is attributed to mutations in the chromosomal *folP* gene, which encodes dihydropteroate synthase (DHPS). Several mutations, such as F_17_L, S_18_L and T_51_M in the *folP* gene, have been shown to confer high-level resistance to sulfamethoxazole, while secondary mutations such as E_208_K and KE_257__dup have been associated with increased MIC values.^40^

In this study, trimethoprim/sulfamethoxazole resistance was predominantly mediated by the *dfrG* gene, with 93% of resistant isolates carrying this determinant. In one isolate, the *dfrG* gene was inserted downstream of the *lukS* and *lukF* loci, which encode PVL (Figure 3). Alignment of the *dfrG* gene loci indicated that *dfrG* was often located near virulence loci. However, this does not provide any evidence of co-selection or regulatory patterns, as no such relationship was observed. The *dfrG* gene is classified as a transposable element, capable of integrating into multiple loci within the bacterial chromosome.^41,42^ This genetic mobility likely explains its detection at various chromosomal sites in the *S. aureus* isolates examined in this study and may underlie its predominance as the principal acquired resistance mechanism to trimethoprim.^43^ The abundance of *dfrG* as the principal resistance mechanism to trimethoprim aligns with previously published data.^30,43^ Resistance to sulfamethoxazole was attributed to mutations in the *folP* gene, with the F_17_L mutation being the primary driver. However, in most strains, the exact mechanism of sulphonamide resistance could not be determined.

While this study offers valuable insights, the generalizability of its findings may be limited by the small sample size, the focus on three hospitals in Lagos State and reliance on samples collected in 2017. Broader, more recent studies encompassing additional regions and including environmental and animal sampling are needed to understand MRSA diversity and transmission in Nigeria. Despite these limitations, the scarcity of genomic data on MRSA in the region underscores the significance of this study as a critical contribution to efforts addressing AMR.

Our findings reveal a high prevalence of MDR MRSA in Lagos State, Nigeria, largely driven by the globally disseminated, high-risk ST772 Bengal Bay clone. The detection of emerging MDR MRSA lineages further underscores the dynamic and evolving nature of MRSA epidemiology in the region. Strengthening genomic surveillance, alongside coordinated intervention strategies, is essential in LMIC to mitigate the expanding threat posed by MRSA and reduce its burden on public health.

## Supplementary Material

dlaf161_Supplementary_Data

## Supplementary data

Figures S1 and S2 and Tables S1–S4 are available as Supplementary data at *JAC-AMR* Online.
