# Supplementary material for: Molecular landscape of methicillin-resistant Staphylococcus aureus strains in clinical infections from hospitals in Lagos, Nigeria
Source: JAC Antimicrob Resist. 2025 Sep 19;7(5):dlaf161. doi: 10.1093/jacamr/dlaf161 (PMC12449137; doi:10.1093/jacamr/dlaf161)
Supplement: dlaf161_Supplementary_Data [file dlaf161_supplementary_data.zip › Olalekan_et_al_Supplementary_1_JAC-AMR_CLEAN.docx]

**Table S1. Characteristics of the isolates in the hospitals**

| Characteristics | Hospital A  46 (43%) | Hospital B  22 (20.6%) | Hospital C  39 (36.4%)39 | Total  107 (100%) |
| --- | --- | --- | --- | --- |
| Sample type  Wound swab/Pus  Urine/catheter tip  HVS/ECS  Aspirate/ulcer/abscess  Blood culture  Ear swab  Trachea/sputum  Eye discharge | 36 (33.6%)  6 (5.6%)  1 (0.9%)  3 (2.8%)  -  -  -  - | 14 (13.1%)  1 (0.9%)  2 (1.9%)  1 (0.9%)  1 (0.9%)  2 (1.9%)  1 (0.9%)  - | 15 (14%)  1 (0.9%)  1 (0.9%)  3 (2.8%)  15 (14.0%)  2 (1.9%)  1 (0.9%)  1 (0.9%) | 65 (60.7%)  8 (7.5%)  4 (3.7%)  7 (6.5%)  16 (15%)  4 (3.7%)  2 (1.9%)  1 (0.9%) |
| Sex  Male  female | 30 (28%)  16 (15%) | 12 (11.2%)  10 (9.3%) | 21(19.6%)  18 (16.8) | 63(*58.9%)*  44 (41.1%) |

HVS-High Vaginal Swab, ECS- Endocervical Swab

**Table S2. Antibiotic resistance profile**

|  |  | resistant isolates | |  |
| --- | --- | --- | --- | --- |
| Category | **Antibiotic** | **MIC range**  **Mg/L** | **Number of resistant isolates (107)** | **Percentage** |
| Penicillins | Benzylpenicillin  oxacillin | >=0.5  2->=4 | 101  61 | 94.4%  57% |
| Lincosamides | Clindamycin | *0.25 - >=4 | 11 | 10.3% |
| macrolides | Erythromycin | >=8 | 51 | 48% |
| Tetracycline | Tetracycline | 2 - >=16 | 47 | 44% |
| Lipopeptides | Daptomycin | 2 | 2 | 1.9% |
| Trimethoprim-sulfamethoxazole | Trimethoprim-Sulfamethoxazole | 80 - >= 360 | 93 | 87% |
| Rifamycin | Rifampicin | >=4 - >=8 | 4 | 3.7% |
| phosphonic | Fosfomycin | 64 - >=128 | 6 | 2.8% |
| Steroid antibacterials | Fusidic acid | 2-4 | 3 | 2.8% |
| Aminoglycosides | Gentamicin | 4 - >=16 | 55 | 51% |
| Fluoroquinolones | Levofloxacin | 4 - >=8 | 51 | 48% |
| Cefoxitin Screening (positive) | Cefoxitin |  | 63 | 59% |

**
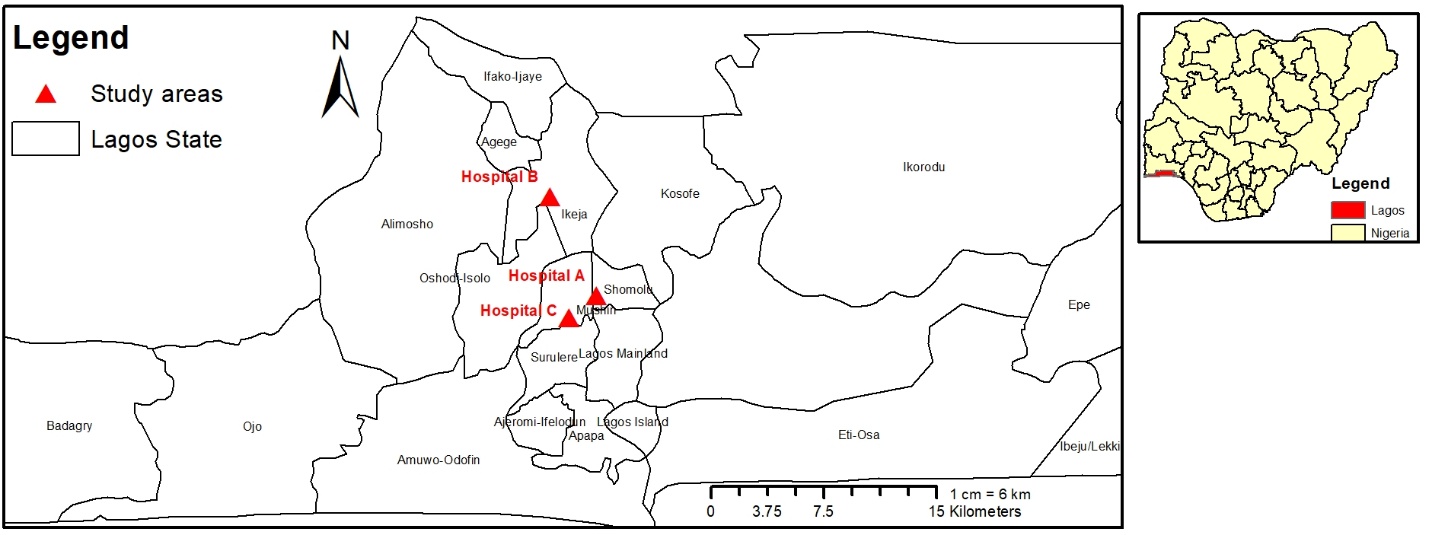
**

**Figure S1. Sketch Map of Lagos State, Nigeria, indicating the study site.**

The study site Hospital A (Lat 6° 30' 33.228'' N; Log 3° 22' 10.5744'' E), Hospital B (6° 35' 44.772'' N; Log3° 20' 13.488'' E), Hospital C (Lat 6° 31' 23.556'' N; Log 3° 20' 30.12'' E),


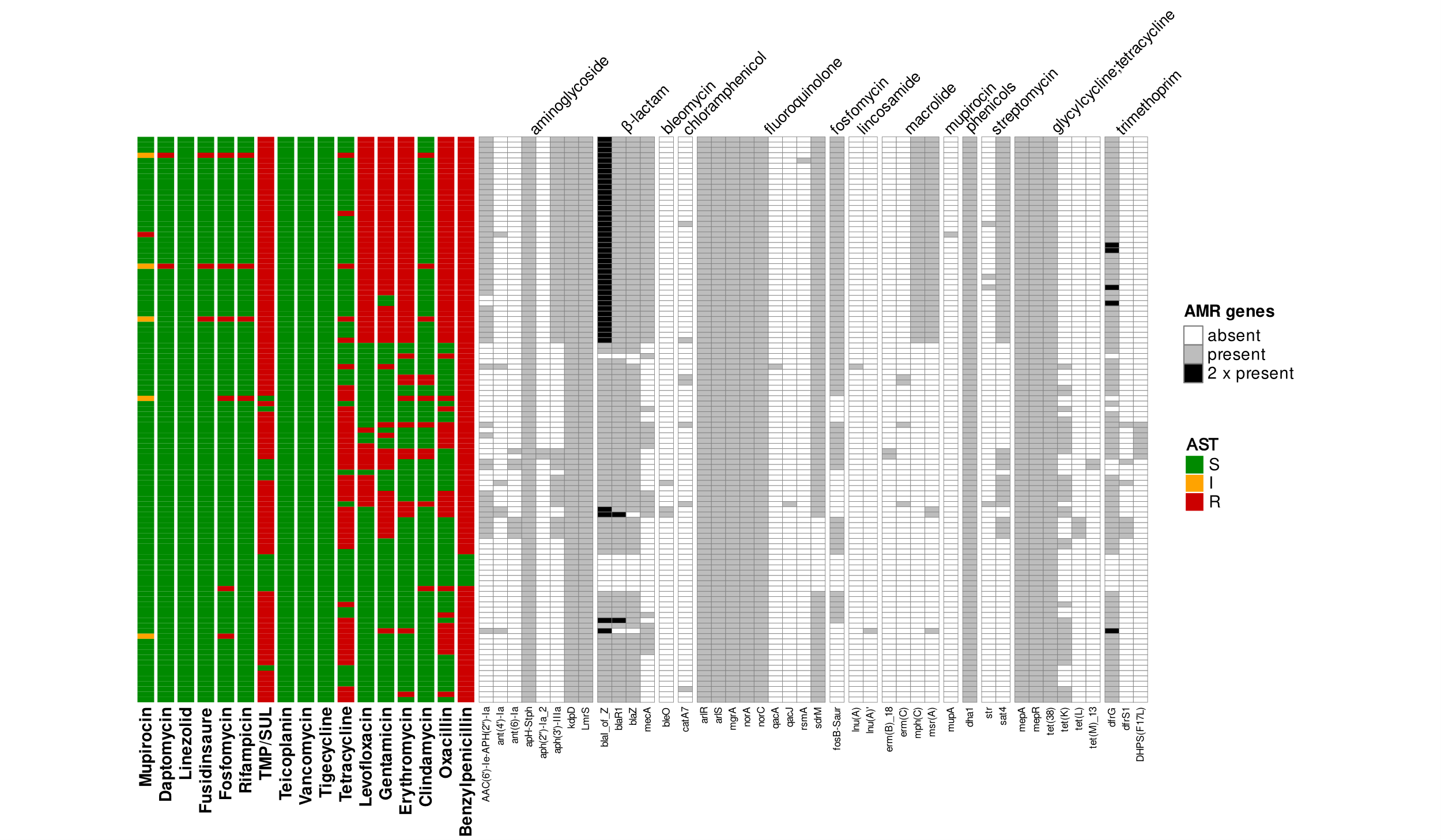


**Figure S2: Overview of phenotypic and genotypic resistance.**

Antibiotic susceptibility is interpreted according to the EUCAST clinical breakpoints. Resistant genes are grouped by antibiotic classes. Abbreviations: S=susceptible, I=susceptible, high exposure, R=resistant.
